# Lung Cancer and Pleural Mesothelioma Risk Assessment for the General Population Exposed to Asbestos in Different Regions of Tehran, Iran

**DOI:** 10.34172/jrhs.2022.98

**Published:** 2022-12-29

**Authors:** Nafiseh Nasirzadeh, Zahra Soltanpour, Yousef Mohammadian, Bahman Pourhasan

**Affiliations:** ^1^Department of Occupational Health Engineering, School of Public Health, Tehran University of Medical Sciences, Tehran, Iran; ^2^Department of Occupational Health Engineering, Faculty of Health, Tabriz University of Medical Sciences, Tabriz, Iran

**Keywords:** Asbestos, Lung, Mesothelioma, Neoplasms, Quantitative health risk assessment

## Abstract

**Background:** Asbestos is a natural fiber leading to health risks like chronic lung diseases. The current study aimed to estimate pleural mesothelioma and lung cancer risk for population exposure to asbestos in Tehran, Iran.

**Study Design:** A cross-sectional study.

**Methods:** According to the annual report of Air Quality Control Company (AQCC), from 2011-2020, carcinogenic risk and mesothelioma were assessed based on the Environmental Protection Agency (EPA) method using the Monte Carlo simulation (MCS). The relative risk (RR) of mortality cancer was calculated based on Camus and colleagues’ model. Moreover, mesothelioma risk was estimated by Bourgault and colleagues’ model.

**Results:** The mean concentration and health risk of asbestos in ambient air generally reduced from 2011 to 2020. The highest mortality risk for lung cancer was 8.4 per 100000 persons in 2011 and reduced to 1.8 in 2017. For mesothelioma, the corresponding values were 8.96 per 100000 persons in 2011 and reduced to 1.92 in 2017.

**Conclusion:** The findings of this study could be helpful to health policymakers in the management of asbestos risk.

## Background

 Asbestos is a trading name referring to a group of six natural fibrous silicate minerals^1^ with exclusive chemical and physical characteristics, such as resistance to heat,^2^ fire, acid, and water, as well as high-tensile strength.^3^ Asbestos has many applications in some industries, such as automobile brake and clutch cement, sheet manufacturing,^4^ and building.^5^ In the Asian-Pacific countries, asbestos was wieldy used due to rapid economic development.^6^ In these countries, a lack of public awareness of the hazards of asbestos leads to adverse health effects.^6^ Inhalation of asbestos in occupational or environmental exposure results in bronchogenic carcinoma,^7^ mesothelioma,^8^ pneumonia,^9^ chronic obstructive pulmonary disease,^10^ and asthma.^11^ Although these effects have been evidenced mainly in workers, they have also been demonstrated in populations non-occupationally exposed to asbestos.^12^

 Currently, Asian countries face high external pressure to confront diseases associated with asbestos.^13^ Asbestos was wieldy used in Asian countries in the Middle East due to the existence of oil and gas.^14^ Nonetheless, several middle east countries, such as Iran, Turkey, Egypt, and Saudi Arabia, have restricted the use of asbestos.^15^ In 2010 and 2011, the use of asbestos was legally banned in Iran^16^; however, asbestos exposure has been reported in Iran.^17^ Reports show that in Tehran, the average concentration was 3.4 × 10^-3^ the phase contrast microscopy (PCM) f/mL (0.1 SEM f/mL), which is considerably higher (more than 68- and 45-fold) than the levels of asbestos reported in outdoor air in the USA and the urban environment of Europe (5 × 10^-5^ PCM f/mL and 2.2 × 10−3 SEM f/mL).^18-20^ Asbestos has been used in buildings for several years, and the erosion or demolition of an old building can lead to the contamination of ambient air with asbestos fibers.^21^

 Health risk assessment is a suitable method for identifying and predicting the health hazard to make-decision about controlling adverse health effects.^22^ In this regard, efforts have been made to provide information about lung cancer risk assessment and mesothelioma in the general population exposed to outdoor and indoor air. For instance, Bourgault et al conducted a study for “population-specific” cancer data. They pointed out that lung cancer and mesothelioma risk is close to 1 per 100 000.^23^ Nowadays, there is some evidence of the incidence of mesothelioma in non-occupational or environmental exposure.^8,24^ Nevertheless, there is no information about predicting the risk of mesothelioma mortality among the general population in Iran. Therefore, the present study aimed to estimate lung cancer and pleural mesothelioma risk assessment for the general population exposed to asbestos in different regions of Tehran, Iran.

## Methods

 The results of asbestos monitoring in ambient air of the different regions of Tehran during the years 2011-2020 were obtained from Tehran Air Quality Control Company (AQCC). According to AQCC’s report, this company conducts several samplings per year in various areas of Tehran and analyses samples according to NIOSH 7400 to assess airborne asbestos fibers. Finally, the annual concentration report is presented. The sample size is different for every region due to traffic volume and geographical area. They are chosen according to the opinion of the AQCC’s experts. The general approach involved the determination of the dose-response relationship. Primely, the range of carcinogenicity risk of asbestos was determined. Thereafter, the relative risk (RR) for lung cancer and mesothelioma was calculated.

###  Lung cancer risk assessment

 Due to the carcinogenicity of asbestos, the health risk assessment was conducted based on the cancer risk using the Environmental Protection Agency (EPA) method.^25,26^ According to the EPA method, mean concentrations for each year were used to estimate exposure concentration (f/mL). Cancer risk was calculated using equations 1 and 2.


Equation (1) EC=C×ET×EF×ED/AT

Equation (2) ELCR=EC×IUR

In equation 1, EC is exposure concentration (f/mL); C signifies the concentration of asbestos (f/mL); ET denotes exposure time (24 hours); EF is exposure frequency (180 days/years)^27^; ED means exposure duration (24 years for adults); and AT is the average time ( $74\;year\times 365\frac{day}{year}\times 24\;hour/day$ )^28^. In equation 2, ELCR is the estimation of the carcinogenicity risk of asbestos; EC signifies exposure concentration (f/mL); and IUR is inhalation unit risk (2.3 × 10^-1^ (f/mL)).^29^ Inhalation (IUR) is an estimate of the lifetime cancer risk associated with inhalation exposure to a concentration of 1 g/m^3^. The IUR can be multiplied by an assessment of lifetime exposure (in µg/m^3^) to estimate the lifetime cancer risk.^30^

 The range of carcinogenicity risk of asbestos is considered 1E^−6^ to 1E^−4^ by EPA guidelines. When ELCR is more than 1 × E ^-4^, the cancer risk is at a considerable level; nonetheless, if ELCR is lower than 1 × E ^-6^, cancer risk is at the acceptable level.^25^ In this study,theMonte Carlo simulation (MCS) method was used for the simulation of the results of cancer risk.^31,32^ MCS is a probabilistic estimation and one of the most common methods used to consider uncertainties related to many risks. In this line, the Crystal Ball software (Version 11.1. USA, Inc) was used. A total of 10 000 repetitions were used to determine variances of ELCR by included variables. A percentile of 95% of ELCR of asbestos was considered the benchmark cancer risk. According to Bourgault and colleagues method,^23^ the predicted RR for lung cancer was estimated as follows:


Equation (3) $$RR=1+K_{L}\times X$$

In this formula, K_L_ is the retained potency factor for lung cancer. “Potency factors” for asbestos in causing lung cancer (KL) and mesothelioma (KM) were derived by fitting mathematical models to data from the studies of occupational cohorts and studies published in EPA. It has been updated several times since 2008 and is based on such characteristics as relative toxicological outcomes, relative metabolic rates, relative absorption rates, quantitative structure-activity relationship methods (SARs), or receptor binding characteristics.^23^ Silverstein et al. reported that for more than 25 years, great efforts were made to develop asbestos risk assessment methods that provide reasonably valid and reliable information.^33^ Moreover, X is the cumulative exposure. Since asbestos has a 20–50 year incubation period, it was calculated for 50 years.^34,35^ Bourgault et al used an approach developed by Nicholson in 1986 for the U.S. EPA to predict the validity of the potency factor. In brief, Nicholson characterized the risks of these asbestos-related cancers from epidemiological studies performed on workers. Thereafter, a linear dose-response relationship was assumed for lung cancer and mesothelioma, respectively.^36^ Predictive validity of the potency factors demonstrated that it has good validity; therefore, we used this K_l_ and K_M_ in our study. Based on potency factors calculated in the study by Bourgault et al, the best estimate (BE), as well as the upper bound (UB) of the uncertainty interval, was retained on this K_L _(BE: 0.0030 and UB:0.011 per unit exposure– i.e. (f/mL*year)^−1^).^23^

 The cumulative lifetime risk (per 100 000 persons) was calculated by the average lifetime exposure concentration of asbestos (C_avg_;f/mL) and the relevant lifetime unit risk (UR; (f/mL)^−1^) as follows:


Equation (4) $$R=C_{avg}\times UR$$

The average lifetime exposure concentration (C_avg_) was calculated as the weighted average outdoor exposure concentration. The lifetime inhalation UR was estimated based on Bourgault’s method. Considering UR (f/mL)^−1^ for lung cancer, the values were obtained at BE: 0.0030 and UB: 0.011.^23^

###  Mesothelioma risk assessment

 We estimated the predicted number of incident cases of mesothelioma (I’M) as a function of the I_M_;


Equation (5) $$I_{M}=\frac{I_{M-Ca}\times K_{M-sel}}{K_{M-Nic}}$$

Where I_M-Ca_ is Nicholson’s K_M_, and K_M-sel_ is the ratio of our K_M_ over Nicholson’s K_M _(K_M-Nic_).^37^ In this formula, K_M _is the retained potency factor for mesothelioma. Since amphiboles have a potency to induce mesothelioma several hundred times greater than that of chrysotile, the potency factor for mesothelioma is more sensitive than other asbestos fibers.^38^ According to AQCC reports, chrysotile, tremolite, and actinolite are the main fibers identified in the air of Tehran city. Therefore, we considered K_M_ for amphibole fibers based on Berman and Crump’s data.^39^ Moreover, BE and UB of K_M_ value were considered 0.021 and 0.065 × 10−8 (f/mL*year)^−1^), respectively. The cumulative lifetime risk (per 100 000 persons) was calculated by Equation (4). In addition, UR (f/mL)^−1^ for lung cancer was considered equal to BE: 0.0032 and UB: 0.0099.^23^

## Results

 As evidenced by the results of the present study, the mean concentration of asbestos in the ambient air was higher than the recommended standard by WHO (5 × 10^-5^ PCM f/mL).^40^ According to Table 1, in general, the mean concentration of asbestos has reduced from 2011-2020 based on AQCC. There was an unexpected increase in exposure to asbestos in 2016, and after that year, the concentration fluctuated. The results of the carcinogenic risk assessment demonstrated that the mean carcinogenic risk was between 1.26E-4 and 9.55E-5.

**Table 1:** Predicted relative risk and mean of lung carcinogenic risk of asbestos in ambient air in Tehran

**Years**	**Mean exposure (f/mL)**	**Cultivated exposure for 50 years**	**Relative risk**		**Mean concentration**	**Lifetime mortality risk** ^a^		**Carcinogenic risk** ^b^
			**BE **	**UB **		**BE **	**UB **	
2011	0.0065	0.3250	1.009	1.030	0.0280	8.4	30.8	2.41 E-4
2012	0.0033	0.1650	1.005	1.010	0.0140	4.2	15.4	1.26 E-4
2013	0.0021	0.1050	1.003	1.010	0.0090	2.7	9.9	7.76 E-5
2014	0.0017	0.0850	1.002	1.009	0.0070	2.1	7.7	6.18 E-5
2015	0.0018	0.0900	1.003	1.009	0.0070	2.1	7.7	6.55 E-5
2016	0.0026	0.1300	1.004	1.010	0.0110	3.3	12.1	9.55 E-5
2017	0.0014	0.0700	1.002	1.007	0.0060	1.8	6.6	5.08 E-5
2018	0.0018	0.0900	1.003	1.009	0.0078	2.3	8.6	6.62 E-5
2019	0.0022	0.1100	1.003	1.012	0.0096	2.9	10.6	8.09 E-5
2020	0.0019	0.0935	1.003	1.001	0.0081	2.4	8.9	6.88 E-5

BE, best estimate; UB, upper bound. ^a^ Lifetime mortality risk (per 100 000 persons). ^b^ Mean of carcinogenic risk of asbestos by Monte Carlo simulation.

 As illustrated in Table 1, cancer risk was considerable during 2011 and 2012. Thereafter, it was reduced after 2013. In 2016, the mean carcinogenic risk increased again. The results of this study indicated that the highest mortality risk for lung cancer was 8.4 per 100 000 persons in 2011. It reduced to 1.8 in 2017 (Table 1). For mesothelioma, the corresponding numbers were 8.96 per 100 000 persons in 2011 and reduced to 1.92 in 2017 (Table 2).

**Table 2:** Incident cases of mesothelioma and lifetime mortality risk for mesothelioma in ambient air in Tehran

**Years**	**Mean** **exposure (f/mL)**	**Cultivated exposure for 50 years**	**Mesothelioma incidence ** ^a^		**Concentration of asbestos ** ^b^	**Lifetime mortality risk** ^c^	
			**BE**	**UB **		**BE **	**UB **
2011	0.0065	0.3250	6.80	21.12	0.0280	8.96	27.70
2012	0.0033	0.1650	3.46	10.72	0.0140	4.48	13.80
2013	0.0021	0.1050	2.20	6.82	0.0090	2.88	8.90
2014	0.0017	0.0850	1.80	5.50	0.0070	2.24	6.90
2015	0.0018	0.0900	1.90	5.85	0.0070	2.24	6.90
2016	0.0026	0.1300	2.70	8.45	0.0110	3.52	10.80
2017	0.0014	0.0700	1.50	4.55	0.0060	1.92	5.90
2018	0.0018	0.0900	1.89	5.85	0.0078	2.50	7.70
2019	0.0022	0.1100	2.31	7.15	0.0096	3.07	9.50
2020	0.0019	0.0935	1.96	6.07	0.0081	2.60	8.02

BE, best estimate; UB, upper bound. ^a^ Incident cases of mesothelioma for various cumulative exposure estimates. ^b^ average lifetime exposure concentration of asbestos. ^c^ Lifetime mortality risk (per 100,000 persons).

## Discussion

 The results of the current study pointed out that the mean concentration of asbestos in ambient air was higher than the recommended standard by WHO in 1998 (5 × 10^-5^ PCM f/mL)^40^. Not only AQCC reports but also the results of previous studies demonstrated that the mean concentration of asbestos was higher than standard in urban and traffic areas in Tehran.^14^ Although since 2010, the asbestos prohibition regulation was implemented,^41^ many studies suggested that asbestos still exists in ambient air in Iran.^42^ For example, the study by Maleki et al suggested that from 2018-2019, the mean concentrations of asbestos were reported as 0.0023 ± 0.013 fiber/mL in the cold season and 0.0014 ± 0.0007 fiber/mL in the warm season in ambient air of Tehran.^43^

 It is natural that seasonal variables can be effective in changing the concentration of air pollutants. One of the important reasons for seasonal variables is wind direction, which can be effective during sampling and accounts for the fluctuations observed in the concentration of asbestos. In addition, our previous study on the concentration and cancer risk of asbestos in Middle East countries illustrated that the concentration of asbestos in the outdoor air of traffic, urban, and rural subgroups in Iran were 0.021 f/mL, 0.006 f/mL, and 0.017 f/mL, respectively.^44^ This study suggested that among the Middle Eastern cities and countries, the highest concentration of asbestos pertained to Tehran, Iran. The results of previous studies suggest that not only asbestos is present in the ambient air of Tehran, but also the concentration of asbestos is higher than the recommended standard by WHO.

 The mean concentration of asbestos has reduced from 2011-2020 based on AQCC, and there was an unexpected increase in exposure to asbestos in 2016 with no clear reason. Nonetheless, it seems that the importation of asbestos-containing products has played an effective role. For instance, although since 2010, the importation of asbestos-containing products is prohibited unless the importer has a permit for the shipment issued by the Environmental Protection Organization, in 2016, the head of the railway announced the importation of asbestos-containing wagons from Europe to Iran. After that year, concentration fluctuated in a decreasing manner. Although the reason for these fluctuations is not clear, it seems that decreased concentration has been slightly affected by this ban law. However, the justification plan for asbestos importation by different industries, such as automotive brake and clutch, as well as asbestos cement pipes, has been able to import million tonnes of asbestos products into the country.

 The results of the carcinogenic risk assessment illustrated that the mean carcinogenic risk was between 1.26E^-4^ and 9.55E^-5^. In addition, cancer risk was considerable during 2011 and 2012; thereafter, it reduced after 2013. In 2016, the mean carcinogenic risk increased again. Tavakoli et al reported that the carcinogenic risk was between 2.42E-5 (minimum risk) and 1.13E-3 (maximum risk) for smokers, as well as 2.86E-6 (minimum risk) and 1.13E-3 (maximum risk) for nonsmokers in 2016.^45^ Maleki et al reported that carcinogenic risk was between 3.78E−4 and 2.65E−3 and from 5.67E−4 to 3.97E−3 for warm and cold seasons, respectively, from 2018 to 2019.^43^ Given that the AQCC reports the mean concentration for the whole city, the difference in results is to be expected. Similar conditions were reported in European Union. In Poland, the maximum carcinogenic risk was higher than 1E-04, and the highest asbestos concentration was estimated in town centers.^21^ In Ruda Śl City, it was calculated at 3.68E-4.^21^ Although in 1991, European Union members banned the use of asbestos,^46^ the carcinogenic risk was considerable after 22 years in Poland. In addition, Alpert et al. reported mesothelioma incidence after 40 years.^46^

 The results of this study show that the highest mortality risk for lung cancer was 8.4 per 100 000 persons in 2011. It reduced to 1.8 in 2017. Bourgault et al reported that the lifetime mortality risk ranged from 0.7 and 2.6 per 100 000 persons continuously exposed to asbestos for 80 years in Canada.^23^ The comparison of the results shows that lung cancer risk in Tehran is higher than that in Canada. However, in both two studies, these numbers have exceeded the health threshold for considering a lifetime cancer risk negligible (i.e., 1 per 100 000 ).

 For mesothelioma, the corresponding number was 8.96 per 100 000 persons in 2011 and reduced to 1.92 in 2017. In the study by Bourgault et al, lifetime mortality risk for mesothelioma ranged from 0.7 and 2.3 per 100 000 persons.^23^ Since mesothelioma risk assessment for a population environmentally exposed to asbestos is low, we could not compare our results to those reported in similar studies. Since mesothelioma risk assessment is dependent on K_M_, it may differ in various studies; therefore, updated epidemiological data can affect risk assessment.

 In 2010 and 2011, the use of asbestos was legally banned in Iran^16^; nevertheless, asbestos exposure has been reported in Iran.^17^ Asbestos has been used in buildings for several years. The erosion or demolition of an old building can lead to the contamination of ambient air with asbestos fibers^21^. According to Asbestos Convention (in 1986) at the International Labour Organization (ILO) (No. 162), measures should be implemented to observe safety in the use of asbestos and control exposure to asbestos. Currently, in Iran, the prohibition of the use of asbestos and replacement of asbestos with other less harmful materials is observed in industrial settings.

 Among the notable limitations of this study, we can refer to the fact that risk assessment has been calculated based on the available data in AQCC. Although, according to the Supreme Council for the Protection of Environment since October 2011, AQCC has been obligated to regularly sample and analyze airborne fibers, the analyzing strategy of the company (using PCM approach) cannot exactly clarify the efficacy of mentioned forbiddance in asbestos concentrations in urban zones. Therefore, appropriate sampling and analyzing methods should be taken to obtain reliable results. Furthermore, according to EPA method, risk assessments are conducted using PCM data, and concentration has a main role in calculations. Cancers, such as mesothelioma, not only depend on the concentration of asbestos, but also other main factors such as genetics, food, and smoking are significant interveners.

## Conclusion

 The results of the present study indicated that the mean concentration of asbestos was higher than the recommended standard by WHO. In 2011 and 2012, the carcinogenic risk from asbestos exposure was at a considerable level. Moreover, the lifetime mortality risk for lung cancer and mesothelioma risk has exceeded the health threshold. Although estimates show that due to decreased concentration of asbestos, the risk has decreased in recent years, the mean concentration of asbestos in ambient air is still higher than the recommended standard by WHO. It seems that decreased concentration has been slightly affected by this ban law. However, asbestos importation by different industries, such as automotive brake and clutch, as well as asbestos cement pipes, play an important role in releasing asbestos into the air. The finding of this study could be helpful to health policymakers in the management of asbestos risk.

**Highlights**

- The present study aimed to estimate pleural mesothelioma and lung cancer risk for population exposure to asbestos in Tehran, Iran.
- The mean concentration of asbestos in ambient air generally reduced from 2011-2020.
- The highest mortality risk for lung cancer was 8.4 per 100000 persons in 2011. It reduced to 1.8 in 2017.
- For mesothelioma, the corresponding numbers were 8.96 per 100000 persons in 2011, and it also reduced to 1.92 in 2017.

## Acknowledgments

 We would like to express our special thanks to the people who facilitated data collection. This research behind it would not have been possible without the special support of our good colleague, Mr. Hossein Hassankhani in Air Quality Control Compony, Tehran, Iran. His enthusiasm, knowledge and exacting attention to detail have been inspiring.

## Conflicts of interest

 The authors declare that they have no conflict of interest.

## Ethical approval

 The study was based on a literature search and was not an experimental study; therefore, it did need ethics committee approval.

## Funding

 No funding was received.
